# Editorial: Non-invasive biomarkers for sperm retrieval in non-obstructive patients

**DOI:** 10.3389/fendo.2024.1476514

**Published:** 2024-09-26

**Authors:** Marjan Sabbaghian, Yanhe Lue

**Affiliations:** ^1^ Department of Andrology, Reproductive Biomedicine, Research Center, Royan Institute for Reproductive Biomedicine, Academic Center for Education, Culture, and Research (ACECR), Tehran, Iran; ^2^ Division of Endocrinology, Department of Medicine, The Lundquist Institute at Harbor-University of California Los Angeles (UCLA) Medical Center, Los Angeles, CA, United States

**Keywords:** male infertility, non-obstructive azoospermia, micro-TESE, seminal plasma, biomarkers

Azoospermia, characterized by the lack of sperm in the ejaculate, affects 1% of men in general population and accounts for about 10%–15% of infertile men. Azoospermia is generally classified as obstructive azoospermia (OA) due to male reproductive tract obstruction and nonobstructive azoospermia (NOA) due to genetic or acquired spermatogenesis defects. NOA is the most severe type and most difficult to treat form of male infertility.

Testicular sperm extraction (TESE) or microdissection TESE (m-TESE) are standard procedures to retrieve spermatozoa for *in vitro* fertilization (IVF) using intracytoplasmic sperm injection (ICSI). However, these minor surgical procedures are not uniformly successful, with outcomes heavily depending on the etiology of the conditions, surgical skills, embryologist or andrologist’s experience in finding spermatozoa in extracted testicular tissues.

In addition to above mentioned variables, it is also essential to distinguish which NOA patient has a good chance of testicular sperm extraction before sperm retrieval surgery. Numerous studies have reported that models based on serum hormones, including FSH, LH, testosterone, and inhibin B, could be useful in predicting the presence of testicular spermatozoa in NOA patients but unfortunately, the specificity and sensitivity of these models are all moderate. A testicular biopsy is a powerful approach to achieving histological evaluation of the human testes, but this technique is invasive and incomplete in assessing majority of seminiferous tubules.

As the editors of this Research Topic, we are pleased to present to you five groundbreaking research papers and up-to-date reviews that have been carefully selected for their significance and impact on the field of male infertility. These papers cover different points of view related to NOA and the latest advancements in technology to important developments in healthcare and beyond. Recent advancements in reproductive medicine have guided novel strategies for addressing male infertility, particularly in cases of non-obstructive azoospermia (NOA).

Fontana et al., conducted a literature review to critically evaluate the genetics of NOA, the current knowledge of molecular non-invasive biomarkers in NOA patients, and their applicability to clinical practice. Special attention is paid to unique genetic profiles associated with spermatogenesis failure, which might be considered as signatures of NOA in a subset of cases. They address the challenge of sperm retrieval in NOA through non-invasive biomarkers. Moreover, they delve into promising perspectives, elucidating innovative approaches grounded in multi-omics methodologies, including genomics, transcriptomics and proteomics. These cutting-edge techniques, combined with the clinical and genetics features of patients, could improve the use of biomarkers in personalized medical approaches, patient counseling, and the decision-making continuum. Finally, Artificial intelligence (AI) holds significant potential in the realm of combining biomarkers and clinical data, also in the context of identifying non-invasive biomarkers for sperm retrieval.

The study by Fietz et al. aimed to discover biomarkers in seminal plasma that could be employed for a non-invasive differential diagnosis of OA/NOA in order to rationalize surgery recommendations and improve success rates. Using label-free LC-MS/MS, they compared the proteomes of seminal plasma samples from fertile men and infertile men diagnosed with OA and NOA with successful sperm retrieval and found the protein expression levels of 42 proteins to be significantly down-regulated in seminal plasma from Sertoli cell-only (SCO) NOA patients relative to health controls (HC) whereas only one protein was down-regulated in seminal plasma from mixed testicular atrophy (MTA) patients. Analysis of tissue and cell expression suggested that the testis-specific proteins LDHC, PGK2, DPEP3, and germ-cell enriched heat-shock proteins HSPA2 and HSPA4L are promising biomarkers of spermatogenic function. Western blotting revealed a significantly lower abundance of LDHC and HSPA2 in the seminal plasma of men with NOA (SCO and MTA) compared to controls.

Shi et al., in their contribution to “MicroRNAs in spermatogenesis dysfunction and male infertility: clinical phenotypes, mechanisms and potential diagnostic biomarkers” provided a comprehensive review on the role of miRNAs in male infertility and spermatogenesis. They presented data related to the effects of miRNAs on spermatogenesis, sperm quality and quantity, fertilization, embryo development, and assisted reproductive technology (ART) outcomes. Also, they summarize the targets of miRNAs and the resulting functional effects that occur due to changes in miRNA expression at various stages of spermatogenesis, including undifferentiated and differentiating spermatogonia, spermatocytes, spermatids, and Sertoli cells (SCs). They conclude that miRNAs are promising biomarkers for diagnosing male infertility and predicting ART outcomes.

Tang et al., using three NOA microarray datasets (GSE45885, GSE108886, and GSE145467), explored the role of core regulatory genes in the pathogenesis of NOA and tried to identify which one are closely associated with azoospermia. They used Differential gene analysis, consensus cluster analysis, and WGCNA and machine learning algorithms to detect potential biomarkers. They identified IL20RB, C9orf117, HILS1, PAOX, and DZIP1 genes as those which possess the strongest association with NOA and could be used as potential therapeutic targets for NOA patients.

Xu et al., in their contribution summarizes the regulatory mechanisms of Fanconi anemia (FA)-related genes in male azoospermia, with the aim of providing a theoretical reference for clinical research and exploration of related genes. There are some evidences that show FA cross-linked anemia (FA) pathway is closely related to the occurrence of NOA. There are FA gene mutations in male NOA patients, which cause significant damage to male germ cells. The FA pathway is activated in the presence of DNA inter-strand cross-links; the key step in activating this pathway is the mono-ubiquitination of the FANCD2-FANCI complex, and the activation of the FA pathway can repair DNA damage such as DNA double -strand breaks. Therefore, FA pathway defect affects germ cells during DNA damage repair, resulting in minimal or even disappearance of mature sperm in males.

In conclusion, a great challenge for the future is the identification of reliable biomarkers for predicting sperm retrieval result that would enable avoiding the invasive procedures. Various types of molecules, such as methylated DNAs, coding or non-coding RNAs, proteins, and genetic features of patients alongside with using cutting-edge techniques and AI maybe useful in this field.

